# Supplementary material for: Composition of terrestrial mammal assemblages and their habitat use in unflooded and flooded blackwater forests in the Central Amazon
Source: PeerJ. 2022 Dec 12;10:e14374. doi: 10.7717/peerj.14374 (PMC9753760; doi:10.7717/peerj.14374)
Supplement: Supplemental Information 1 — *Species that had their occupancy evaluated. §Species considered eventual and not included in the analysis. [file peerj-10-14374-s001.docx]

| **Class/Order/Species** | **Common Name** |  | **Habitat** | | | |  | **Locomotor ^a^** | **Diet ^a^** | **IUCN** | **Group Size ^b^** | **Mean Body Mass (kg) ^b^** |
| --- | --- | --- | --- | --- | --- | --- | --- | --- | --- | --- | --- | --- |
|  |  | ***Terra firme***  **Records (RAI)** | | **Relative Biomass** | ***Igapó***  **Records (RAI)** | | **Relative Biomass** |  |  |  |  |  |
| **MAMMALIA** |  |  |  |  |  |  |  |  |  |  |  |  |
| **Perrisodactyla** |  |  |  |  |  |  |  |  |  |  |  |  |
| *Tapirus terrestris* | South American Tapir * | 17 | (0.28) | 46.93 | 7 | (0.15) | 24.57 | Te | Fr/Hb | VU | 1 | 166 |
| **Artiodactyla** |  |  |  |  |  |  |  |  |  |  |  |  |
| *Mazama americana* | Red Brocket * | 33 | (0.55) | 11.14 | 20 | (0.42) | 8.58 | Te | Fr/Hb | LC | 1 | 20.30 |
| *Mazama nemorivaga* | Brown Brocket Deer * | 108 | (1.80) | 29.46 | 24 | (0.51) | 8.32 | Te | Fr/Hb | LC | 1 | 16.40 |
| *Dicotyles tajacu* | Collared Peccary * | 75 | (1.25) | 310.84 | 38 | (0.80) | 200.21 | Te | Fr/Hb | LC | 11.7 | 21.30 |
| *Tayassu pecari* | White lipped peccary | 1 | (0.02) | 53.79 | - | - | - | Te | Fr/Hb | VU | 103 | 31.40 |
| **Pilosa** |  |  |  |  |  |  |  |  |  |  |  |  |
| *Tamandua tetradactyla* | Collared anteater | 2 | (0.03) | 0.15 | 2 | (0.04) | 0.20 | Sc | In | LC | 1 | 4.65 |
| *Myrmecophaga tridactyla* | Giant anteater * | 16 | (0.27) | 7.42 | 3 | (0.06) | 1.77 | Te | In | VU | 1 | 27.90 |
| **Cingulata** |  |  |  |  |  |  |  |  |  |  |  |  |
| *Dasypus* spp. | Armadillo * | 266 | (4.42) | 30.30 | 7 | (0.15) | 1.01 | Te | In/On | LC | 1 | 6.85 |
| *Priodontes maximus* | Giant armadillo | 3 | (0.05) | 2.13 | - | - | - | Te | In | VU | 1 | 42.70 |
| **Primates** |  |  |  |  |  |  |  |  |  |  |  |  |
| *Saimiri sciureus* ^§^ | Squirrel Monkey | - | - | - | 1 | (0.02) | 0.55 | Ar | Fr/In | LC | 34.9 | 0.75 |
| *Sapajus apella* ^§^ | Brown Tufted Capuchin | - | - | - | 5 | (0.11) | 2.30 | Ar | Fr/On | LC | 7.9 | 2.76 |
| **Rodentia** |  |  |  |  |  |  |  |  |  |  |  |  |
| *Hydrochoerus hydrochaeris* | Capybara | - | - | - | 1 | (0.02) | 10.04 | Se | Fr/Hb | LC | 10 | 47.50 |
| *Cuniculus paca* | Spotted Paca * | 273 | (4.54) | 37.14 | 87 | (1.84) | 15.05 | Te | Fr/Hb | LC | 1 | 8.18 |
| *Dasyprocta leporina* | Red-rumped Agouti * | 463 | (7.70) | 23.25 | 537 | (11.35) | 34.29 | Te | Fr/Gr | LC | 1 | 3.02 |
| *Myoprocta acouchy* | Red Acouchi * | 1723 | (28.65) | 26.94 | 110 | (2.33) | 2.19 | Te | Fr/Gr | LC | 1 | 0.94 |
| *Proechymis* spp. | Spiny-rat * | 382 | (6.35) | 3.24 | 91 | (1.92) | 0.98 | Te | Fr/Gr | LC | 1 | 0.51 |
| *Guerlinguetus aestuans* ^§^ | Guianan Squirrel | 41 | (0.68) | 0.12 | - | - |  | Sc | Fr/On | LC | 1 | 0.17 |
| **Didelphimorphia** |  |  |  |  |  |  |  |  |  |  |  |  |
| *Didelphis marsupialis* | Common Opossum * | 675 | (11.23) | 12.91 | 169 | (3.57) | 4.11 | Sc | Fr/On | LC | 1 | 1.15 |
| *Philander opossum* | Gray Four-Eyed Opossum * | 282 | (4.69) | 2.53 | 97 | (2.05) | 1.11 | Sc | In/On | LC | 1 | 0.54 |
| *Metachirus nudicaudatus* | Brown Four-eyed Opossum * | 1409 | (23.43) | 12.42 | 43 | (0.91) | 0.48 | Te | In/On | LC | 1 | 0.53 |
| *Marmosa* spp.^§^ | Mouse opossum | 30 | (0.50) | 0.18 | - | - | - | Sc | In/On | LC | 1 | 0.36 |
| *Oecomys* spp.^§^ | Arboreal Rice rat | 16 | (0.27) | 0.10 | - | - | - | Ar | Fr/Gr | LC | 1 | 0.38 |
| **Carnivora** |  |  |  |  |  |  |  |  |  |  |  |  |
| *Nasua nasua* | South American Coati * | 22 | (0.37) | 10.26 | 13 | (0.27) | 7.71 | Te | Fr/On | LC | 7 | 3.79 |
| *Eira barbara* | Tayra * | 19 | (0.32) | 1.31 | 20 | (0.42) | 1.75 | Sc | Ca/On | LC | 1 | 4.14 |
| *Pteronura brasiliensis* | Giant otter | - | - | - | 6 | (0.13) | 16.49 | Se | Ca | VU | 5 | 26.00 |
| *Panthera onca* | Jaguar * | 15 | (0.25) | 21.18 | 3 | (0.06) | 5.38 | Te | Ca | NT | 1 | 84.90 |
| *Puma concolor* | Cougar * | 11 | (0.18) | 9.86 | 3 | (0.06) | 3.42 | Te | Ca | LC | 1 | 53.90 |
| *Leopardus pardalis* | Ocelot * | 26 | (0.43) | 5.15 | 13 | (0.27) | 3.27 | Te | Ca | LC | 1 | 11.90 |
| *Leopardus wiedii* | Margay * | 7 | (0.12) | 0.38 | 12 | (0.25) | 0.83 | Sc | Ca | NT | 1 | 3.27 |
| *Puma yaguaroundi* | Jaguarundi * | 8 | (0.13) | 0.92 | 3 | (0.06) | 0.44 | Te | Ca | LC | 1 | 6.88 |

^a^ Paglia et al. (2012) ^b^ Wilman et al. (2014)

Footnotes: Locomotor are defined as Terrestrial (Te); Scansorial (Sc); Arboreal (Ar); Semiaquatic (Se). Diet are defined as Frugivore/Herbivore (Fr/Hb); Frugivore/Granivore (Fr/Gr); Frugivore/Insectivore (Fr/In); Frugivore/Omnivore (Fr/On); Insectivore (In); Insectivore/Omnivore (In/On); Carnivore/Omnivore (Ca/On) and Carnivore (Ca). IUCN categories are defined as Vulnerable (VU); Least Concern (LC); Near Threatened (NT).
