# Supplementary material for: Composition of terrestrial mammal assemblages and their habitat use in unflooded and flooded blackwater forests in the Central Amazon
Source: PeerJ. 2022 Dec 12;10:e14374. doi: 10.7717/peerj.14374 (PMC9753760; doi:10.7717/peerj.14374)
Supplement: Supplemental Information 2 — R2 represents the squared correlation coefficient between the environmental variable and the ordination scores projected onto the arrow (not the axes). Asterisks indicate significant differences according to the envfit function; *p < 0.05, **p < 0.01, ***p < 0.001. [file peerj-10-14374-s002.docx]

| **Species** | **NMDS1** | **NMDS2** | **R^2^** | **Pr (> r)** |  |
| --- | --- | --- | --- | --- | --- |
| *Cuniculus paca* | −0.945 | −0.328 | 0.03 | 0.305 |  |
| *Dasyprocta leporina* | 0.726 | 0.688 | 0.14 | 0.004 | ** |
| *Dasypus* spp. | −0.576 | −0.817 | 0.28 | 0.001 | *** |
| *Didelphis marsupialis* | −0.212 | −0.977 | 0.12 | 0.012 | * |
| *Eira barbara* | −0.803 | 0.596 | 0.03 | 0.216 |  |
| *Hydrochoerus hydrochaeris* | 0.750 | 0.661 | 0.03 | 0.190 |  |
| *Leopardus pardalis* | −0.526 | −0.851 | 0.07 | 0.054 | . |
| *Leopardus wiedii* | 0.999 | −0.035 | 0.14 | 0.003 | ** |
| *Mazama americana* | −0.563 | −0.826 | 0.04 | 0.244 |  |
| *Mazama nemorivaga* | −0.643 | −0.766 | 0.14 | 0.009 | ** |
| *Metachirus nudicaudatus* | −0.467 | −0.884 | 0.34 | 0.001 | *** |
| *Myoprocta acouchy* | −0.766 | −0.642 | 0.40 | 0.001 | *** |
| *Myrmecophaga tridactyla* | −0.686 | −0.728 | 0.06 | 0.111 |  |
| *Nasua nasua* | −0.995 | 0.100 | 0.03 | 0.307 |  |
| *Panthera onca* | −0.533 | −0.846 | 0.04 | 0.214 |  |
| *Dicotyles tajacu* | −0.373 | −0.928 | 0.04 | 0.247 |  |
| *Philander opossum* | −0.949 | 0.315 | 0.00 | 0.864 |  |
| *Priodontes maximus* | −0.846 | −0.533 | 0.05 | 0.154 |  |
| *Proechimys* spp. | −0.739 | −0.673 | 0.08 | 0.052 | . |
| *Pteronura brasiliensis* | 0.784 | −0.621 | 0.03 | 0.266 |  |
| *Puma concolor* | −0.745 | −0.667 | 0.08 | 0.055 | . |
| *Puma yagouaroundi* | −0.114 | −0.993 | 0.04 | 0.185 |  |
| *Tamandua tetradactyla* | −0.076 | 0.997 | 0.01 | 0.633 |  |
| *Tapirus terrestris* | −0.975 | −0.220 | 0.09 | 0.039 | * |
| *Tayassu pecari* | −0.025 | −1.000 | 0.01 | 0.780 |  |
