# Supplementary material for: Composition of terrestrial mammal assemblages and their habitat use in unflooded and flooded blackwater forests in the Central Amazon
Source: PeerJ. 2022 Dec 12;10:e14374. doi: 10.7717/peerj.14374 (PMC9753760; doi:10.7717/peerj.14374)
Supplement: Supplemental Information 3 — Models were ranked with expected predictive accuracy (elpd) values calculated using a leave-one-out cross-validation approach. Δelpd shows the pairwise differences in elpd (relative to the top model). ΔSE is the standard error of Δelpd. K is the effective number of parameters, and weight is the weight of each variable influencing the parameter. Occupancy (ψ) and probability of detection (p) between the unflooded (terra firme) and seasonally flooded (igapó) forests are indicated. [file peerj-10-14374-s003.docx]

| **Species** | **Model** | **elpd** | **K** | **Δelpd** | **ΔSE** | **Weight** |
| --- | --- | --- | --- | --- | --- | --- |
| *Cuniculus paca* | ψ (for.typ) p (eff) | −566.380 | 7.948 | 0.000 | 0.000 | 0.339 |
|  | ψ (for.typ) p (for.typ) | −566.669 | 10.532 | −0.289 | 4.740 | 0.067 |
|  | ψ (for.typ) p (.) | −567.187 | 6.604 | −0.808 | 1.806 | 0.252 |
|  | ψ (.) p (for.typ) | −571.785 | 10.867 | −5.406 | 6.925 | 0.342 |
|  | ψ (.) p (eff) | −572.249 | 6.675 | −5.869 | 3.388 | 0.000 |
|  | ψ (.) p (.) | −573.335 | 5.666 | −6.955 | 3.829 | 0.000 |
|  |  |  |  |  |  |  |
| *Dasyprocta leporina* | ψ (for.typ) p (for.typ) | −844.589 | 9.988 | 0.000 | 0.000 | 0.609 |
|  | ψ (.) p (for.typ) | −845.241 | 9.262 | −0.652 | 1.611 | 0.000 |
|  | ψ (for.typ) p (eff) | −858.919 | 7.127 | −14.329 | 13.744 | 0.044 |
|  | ψ (.) p (eff) | −859.320 | 6.143 | −14.731 | 13.824 | 0.347 |
|  | ψ (for.typ) p (.) | −861.568 | 6.424 | −16.978 | 13.169 | 0.000 |
|  | ψ (.) p (.) | −861.974 | 5.417 | −17.385 | 13.253 | 0.000 |
|  |  |  |  |  |  |  |
| *Myoprocta acouchy* | ψ (for.typ) p (for.typ) | −604.072 | 7.973 | 0.000 | 0.000 | 0.589 |
|  | ψ (for.typ) p (eff) | −625.238 | 7.425 | −21.166 | 15.760 | 0.409 |
|  | ψ (.) p (for.typ) | −634.778 | 7.759 | −30.706 | 4.226 | 0.001 |
|  | ψ (.) p (eff) | −638.516 | 6.525 | −34.443 | 15.877 | 0.002 |
|  | ψ (for.typ) p (.) | −656.236 | 7.538 | −52.163 | 13.902 | 0.000 |
|  | ψ (.) p (.) | −669.555 | 6.573 | −65.482 | 13.933 | 0.000 |
|  |  |  |  |  |  |  |
| *Proechimys* spp. | ψ (for.typ) p (for.typ) | −660.949 | 5.995 | 0.000 | 0.000 | 0.368 |
|  | ψ (.) p (for.typ) | −664.978 | 5.603 | −4.029 | 2.824 | 0.261 |
|  | ψ (for.typ) p (eff) | −668.967 | 5.526 | −8.018 | 7.098 | 0.372 |
|  | ψ (.) p (eff) | −674.787 | 4.509 | −13.838 | 7.253 | 0.000 |
|  | ψ (.) p (eff) | −676.450 | 4.684 | −15.501 | 7.796 | 0.000 |
|  | ψ (.) p (.) | −682.408 | 3.943 | −21.459 | 8.118 | 0.000 |
|  |  |  |  |  |  |  |
| *Mazama americana* | ψ (for.typ) p (eff) | −201.003 | 3.071 | 0.000 | 0.000 | 0.839 |
|  | ψ (for.typ) p (.) | −201.255 | 2.806 | −0.252 | 0.548 | 0.000 |
|  | ψ (for.typ) p (for.typ) | −201.763 | 3.722 | −0.760 | 1.018 | 0.000 |
|  | ψ (.) p (eff) | −201.882 | 2.291 | −0.879 | 1.623 | 0.001 |
|  | ψ (.) p (.) | −202.063 | 1.865 | −1.060 | 1.781 | 0.161 |
|  | ψ (.) p (for.typ) | −203.499 | 3.494 | −2.496 | 1.664 | 0.000 |
|  |  |  |  |  |  |  |
| *Mazama nemorivaga* | ψ (for.typ) p (eff) | −347.810 | 3.867 | 0.000 | 0.000 | 0.661 |
|  | ψ (for.typ) p (.) | −349.944 | 3.383 | −2.133 | 1.333 | 0.000 |
|  | ψ (for.typ) p (for.typ) | −350.178 | 5.352 | −2.367 | 3.090 | 0.000 |
|  | ψ (.) p (for.typ) | −350.948 | 4.426 | −3.138 | 4.472 | 0.339 |
|  | ψ (.) p (eff) | −353.720 | 3.302 | −5.910 | 3.202 | 0.000 |
|  | ψ (.) p (.) | −356.137 | 2.776 | −8.326 | 3.368 | 0.000 |
|  |  |  |  |  |  |  |
| *Tapirus terrestris* | ψ (for.typ) p (.) | −122.552 | 1.762 | 0.000 | 0.000 | 1.000 |
|  | ψ (for.typ) p (for.typ) | −122.937 | 2.253 | −0.385 | 0.475 | 0.000 |
|  | ψ (.) p (for.typ) | −123.204 | 2.200 | −0.653 | 0.541 | 0.000 |
|  | ψ (.) p (.) | −123.265 | 1.326 | −0.714 | 1.004 | 0.000 |
|  | ψ (for.typ) p (eff) | −123.561 | 2.428 | −1.009 | 0.388 | 0.000 |
|  | ψ (.) p (.) | −124.215 | 1.900 | −1.663 | 1.104 | 0.000 |
|  |  |  |  |  |  |  |
| *Dicotyles tajacu* | ψ (for.typ) p (.) | −305.118 | 3.190 | 0.000 | 0.000 | 0.701 |
|  | ψ (for.typ) p (eff) | −305.203 | 3.552 | −0.085 | 0.377 | 0.299 |
|  | ψ (for.typ) p (for.typ) | −306.185 | 4.807 | −1.066 | 0.678 | 0.000 |
|  | ψ (.) p (eff) | −309.301 | 2.764 | −4.182 | 2.719 | 0.000 |
|  | ψ (.) p (.) | −309.662 | 2.676 | −4.543 | 2.769 | 0.000 |
|  | ψ (.) p (for.typ) | −310.867 | 5.245 | −5.748 | 2.720 | 0.000 |
|  |  |  |  |  |  |  |
| *Didelphis marsupialis* | ψ (for.typ) p (eff) | −761.833 | 6.047 | 0.000 | 0.000 | 0.541 |
|  | ψ (for.typ) p (for.typ) | −761.903 | 7.329 | −0.070 | 7.315 | 0.445 |
|  | ψ (for.typ) p (.) | −769.885 | 5.041 | −8.052 | 3.807 | 0.000 |
|  | ψ (.) p (eff) | −773.462 | 5.257 | −11.629 | 3.870 | 0.000 |
|  | ψ (.) p (for.typ) | −773.535 | 6.913 | −11.702 | 8.564 | 0.014 |
|  | ψ (.) p (.) | −781.507 | 4.286 | −19.674 | 5.320 | 0.000 |
|  |  |  |  |  |  |  |
| *Philander opossum* | ψ (for.typ) p (eff) | −498.207 | 6.971 | 0.000 | 0.000 | 0.797 |
|  | ψ (.) p (eff) | −499.283 | 5.890 | −1.075 | 1.935 | 0.000 |
|  | ψ (for.typ) p (.) | −501.513 | 6.141 | −3.306 | 2.061 | 0.000 |
|  | ψ (for.typ) p (for.typ) | −501.636 | 9.260 | −3.429 | 3.139 | 0.000 |
|  | ψ (.) p (for.typ) | −502.496 | 8.379 | −4.289 | 3.963 | 0.203 |
|  | ψ (.) p (.) | −502.801 | 5.377 | −4.594 | 3.006 | 0.000 |
|  |  |  |  |  |  |  |
| *Metachirus nudicaudatus* | ψ (for.typ) p (for.typ) | −618.141 | 9.827 | 0.000 | 0.000 | 0.000 |
|  | ψ (.) p (for.typ) | −619.106 | 7.874 | −0.966 | 2.783 | 0.673 |
|  | ψ (for.typ) p (eff) | −695.435 | 9.093 | −77.294 | 27.408 | 0.327 |
|  | ψ (for.typ) p (.) | −709.311 | 8.376 | −91.171 | 27.394 | 0.000 |
|  | ψ (.) p (eff) | −717.722 | 8.464 | −99.582 | 25.108 | 0.000 |
|  | ψ (.) p (.) | −731.541 | 7.662 | −113.401 | 25.120 | 0.000 |
|  |  |  |  |  |  |  |
| *Myrmecophaga tridactyla* | ψ (for.typ) p (.) | −98.089 | 2.069 | 0.000 | 0.000 | 0.960 |
|  | ψ (for.typ) p (eff) | −98.447 | 2.192 | −0.359 | 0.068 | 0.040 |
|  | ψ (for.typ) p (for.typ) | −98.580 | 2.591 | −0.491 | 0.576 | 0.000 |
|  | ψ (.) p (for.typ) | −99.409 | 2.595 | −1.321 | 1.056 | 0.000 |
|  | ψ (.) p (.) | −100.946 | 1.517 | −2.857 | 1.853 | 0.000 |
|  | ψ (.) p (eff) | −101.191 | 1.662 | −3.102 | 1.868 | 0.000 |
|  |  |  |  |  |  |  |
| *Dasypus* spp. | ψ (.) p (for.typ) | −476.565 | 3.683 | 0.000 | 0.000 | 0.574 |
|  | ψ (for.typ) p (for.typ) | −476.809 | 3.813 | −0.244 | 0.393 | 0.408 |
|  | ψ (for.typ) p (eff) | −491.222 | 4.944 | −14.658 | 6.764 | 0.018 |
|  | ψ (for.typ) p (.) | −493.641 | 4.153 | −17.076 | 6.769 | 0.000 |
|  | ψ (.) p (eff) | −522.268 | 4.642 | −45.703 | 5.050 | 0.000 |
|  | ψ (.) p (.) | −524.331 | 3.536 | −47.767 | 4.978 | 0.000 |
|  |  |  |  |  |  |  |
| *Panthera onca* | ψ (.) p (for.typ) | −95.847 | 2.256 | 0.000 | 0.000 | 0.999 |
|  | ψ (for.typ) p (for.typ) | −95.939 | 2.368 | −0.092 | 0.392 | 0.001 |
|  | ψ (for.typ) p (.) | −96.427 | 2.317 | −0.579 | 0.370 | 0.000 |
|  | ψ (for.typ) p (eff) | −96.862 | 2.531 | −1.015 | 0.505 | 0.000 |
|  | ψ (.) p (.) | −98.002 | 1.756 | −2.155 | 1.566 | 0.000 |
|  | ψ (.) p (eff) | −98.209 | 1.856 | −2.362 | 1.578 | 0.000 |
|  |  |  |  |  |  |  |
| *Puma concolor* | ψ (for.typ) p (for.typ) | −79.479 | 1.727 | 0.000 | 0.000 | 0.844 |
|  | ψ (for.typ) p (.) | −79.507 | 1.498 | −0.028 | 0.453 | 0.037 |
|  | ψ (.) p (for.typ) | −79.587 | 1.694 | −0.108 | 0.314 | 0.000 |
|  | ψ (for.typ) p (eff) | −80.057 | 1.596 | −0.578 | 0.443 | 0.000 |
|  | ψ (.) p (.) | −80.161 | 1.015 | −0.682 | 1.343 | 0.119 |
|  | ψ (.) p (eff) | −80.760 | 1.180 | −1.281 | 1.377 | 0.000 |
|  |  |  |  |  |  |  |
| *Leopardus pardalis* | ψ (for.typ) p (.) | −175.917 | 2.417 | 0.000 | 0.000 | 0.805 |
|  | ψ (.) p (.) | −176.205 | 1.894 | −0.288 | 0.976 | 0.194 |
|  | ψ (.) p (for.typ) | −176.413 | 2.792 | −0.496 | 0.780 | 0.000 |
|  | ψ (for.typ) p (for.typ) | −176.638 | 3.249 | −0.721 | 0.529 | 0.000 |
|  | ψ (for.typ) p (eff) | −176.796 | 2.895 | −0.879 | 0.198 | 0.000 |
|  | ψ (.) p (eff) | −177.004 | 2.324 | −1.087 | 0.981 | 0.000 |
|  |  |  |  |  |  |  |
| *Leopardus wiedii* | ψ (for.typ) p (for.typ) | −98.142 | 1.786 | 0.000 | 0.000 | 0.593 |
|  | ψ (.) p (for.typ) | −98.142 | 1.708 | 0.000 | 0.023 | 0.007 |
|  | ψ (.) p (.) | −98.276 | 1.026 | −0.134 | 1.185 | 0.399 |
|  | ψ (for.typ) p (.) | −98.492 | 1.608 | −0.351 | 0.844 | 0.000 |
|  | ψ (.) p (eff) | −98.693 | 1.157 | −0.551 | 1.195 | 0.000 |
|  | ψ (for.typ) p (eff) | −98.913 | 1.718 | −0.771 | 0.876 | 0.000 |
|  |  |  |  |  |  |  |
| *Puma yagouaroundi* | ψ (.) p (.) | −63.747 | 1.536 | 0.000 | 0.000 | 0.569 |
|  | ψ (.) p (for.typ) | −63.762 | 1.887 | −0.016 | 0.460 | 0.431 |
|  | ψ (for.typ) p (for.typ) | −64.154 | 2.364 | −0.407 | 0.414 | 0.000 |
|  | ψ (for.typ) p (.) | −64.421 | 2.252 | −0.674 | 0.256 | 0.000 |
|  | ψ (.) p (.) | −64.445 | 1.718 | −0.699 | 0.283 | 0.000 |
|  | ψ (for.typ) p (eff) | −65.192 | 2.482 | −1.445 | 0.461 | 0.000 |
|  |  |  |  |  |  |  |
| *Nasua nasua* | ψ (for.typ) p (for.typ) | −145.235 | 3.727 | 0.000 | 0.000 | 0.852 |
|  | ψ (for.typ) p (.) | −148.222 | 2.829 | −2.987 | 3.142 | 0.148 |
|  | ψ (for.typ) p (eff) | −148.936 | 3.288 | −3.701 | 3.283 | 0.000 |
|  | ψ (.) p (.) | −152.298 | 2.439 | −7.063 | 3.432 | 0.000 |
|  | ψ (.) p (eff) | −152.937 | 2.966 | −7.702 | 3.586 | 0.000 |
|  | ψ (.) p (for.typ) | −155.061 | 6.700 | −9.826 | 3.831 | 0.000 |
|  |  |  |  |  |  |  |
| *Eira barbara* | ψ (.) p (eff) | −130.987 | 1.561 | 0.000 | 0.000 | 0.623 |
|  | ψ (for.typ) p (eff) | −131.041 | 2.025 | −0.054 | 0.662 | 0.377 |
|  | ψ (.) p (.) | −131.447 | 1.470 | −0.460 | 0.093 | 0.000 |
|  | ψ (for.typ) p (.) | −131.463 | 1.974 | −0.476 | 0.695 | 0.000 |
|  | ψ (for.typ) p (for.typ) | −132.314 | 3.016 | −1.327 | 0.831 | 0.000 |
|  | ψ (.) p (for.typ) | −132.409 | 2.586 | −1.422 | 0.598 | 0.000 |
