# Supplementary figures and images for: Composition of terrestrial mammal assemblages and their habitat use in unflooded and flooded blackwater forests in the Central Amazon
Source: PeerJ. 2022 Dec 12;10:e14374. doi: 10.7717/peerj.14374 (PMC9753760; doi:10.7717/peerj.14374)

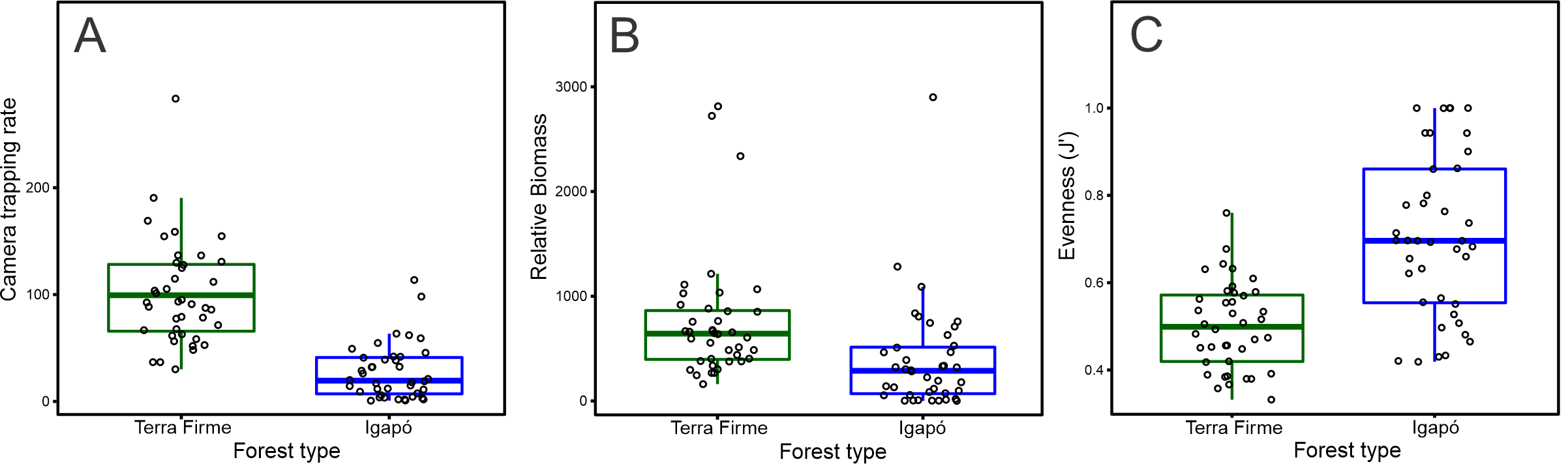

Supplement: Supplemental Information 4 [file peerj-10-14374-s004.png]

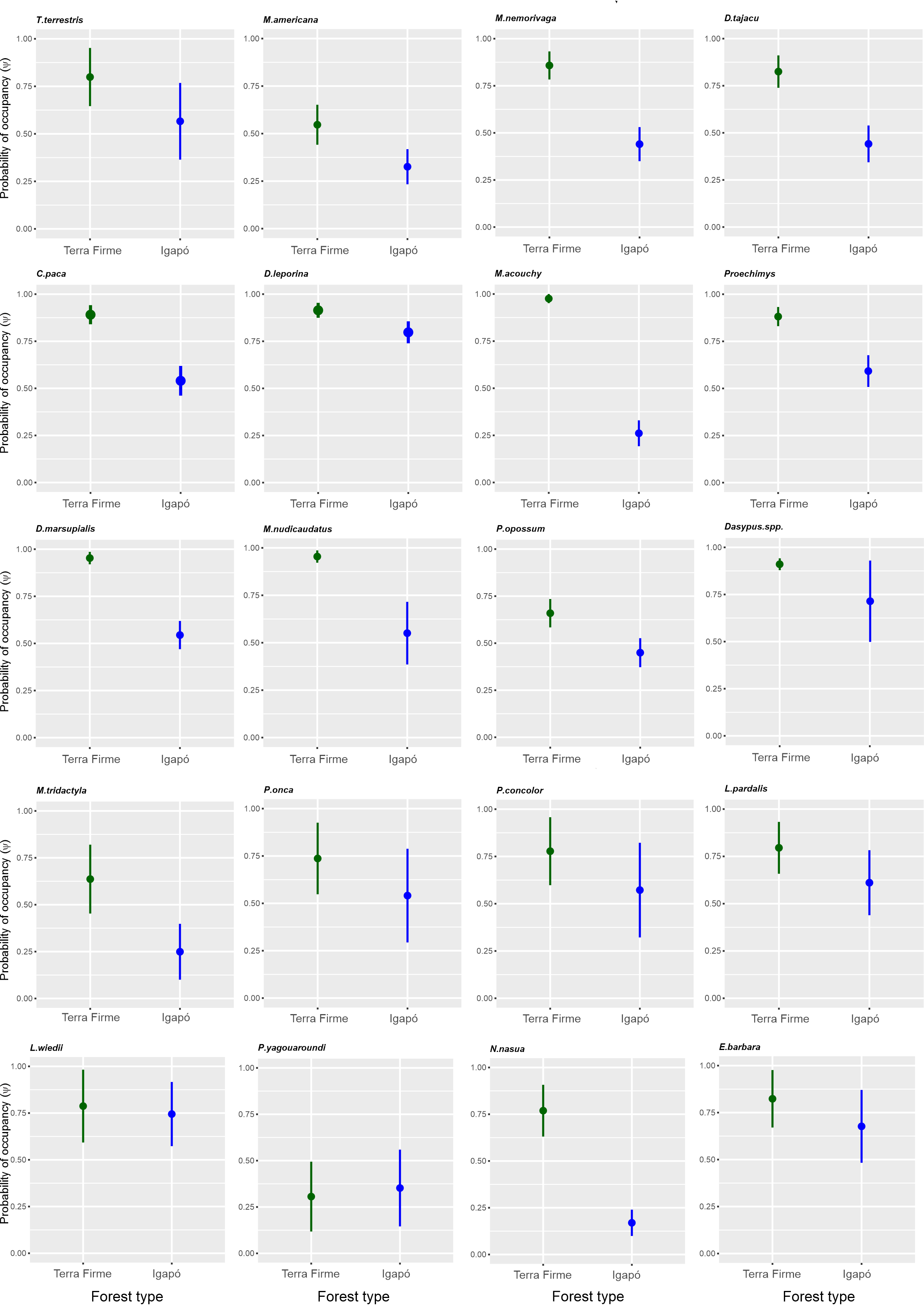

Supplement: Supplemental Information 5 [file peerj-10-14374-s005.png]

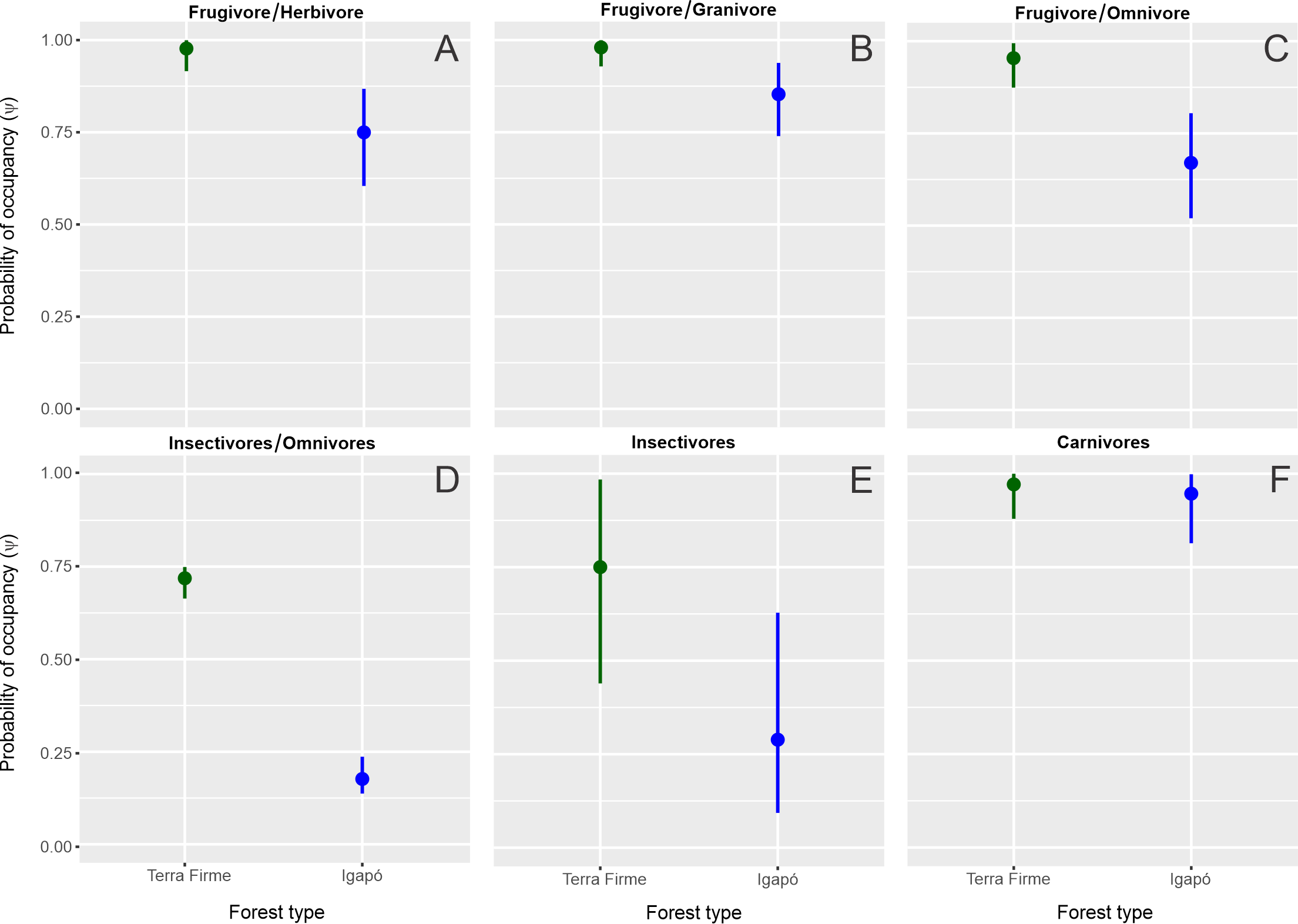

Supplement: Supplemental Information 6 [file peerj-10-14374-s006.png]
